# High-Performance Solid Polymer Electrolyte Constructed from Long-Chain Regulated Random Copolymers and Porous PI Composites

**DOI:** 10.3390/polym18060685

**Published:** 2026-03-11

**Authors:** Qian Zhang, Mingyang Cao, Chenxia Tang, Yuqing Zhou, Xiaoli Peng

**Affiliations:** 1 School of Materials and Energy, University of Electronic Science and Technology of China, Chengdu 611731, China; 202321030322@std.uestc.edu.cn (M.C.); 202211030527@std.uestc.edu.cn (C.T.); 202322030332@std.uestc.edu.cn (Y.Z.); 2 School of Integrated Circuit Science and Engineering (Exemplary School of Microelectronics), University of Electronic Science and Technology of China, Chengdu 611731, China; zq@uestc.edu.cn; 3 Frontier Center of Energy Distribution and Integration, Tianfu Jiangxi Lab, No. 366, Laboratory Road, Chengdu 641419, China

**Keywords:** solid polymer electrolyte, polyester, polyimide support, long-carbon-chain regulation, lithium metal battery

## Abstract

Solid polymer electrolytes (SPEs) hold great potential in high-safety energy storage but face two key bottlenecks: low room-temperature ionic conductivity and insufficient mechanical strength. This study proposes a synergistic optimization strategy of “long-carbon-chain regulation of polymer microstructure combined with porous polyimide (PI) support”. A linear random copolyester, poly(1,3-propylene-co-1,4-butylene succinate-co-sebacate) (PBPSS), was synthesized via melt polycondensation using 1,3-propanediol, 1,4-butanediol, succinic acid, and sebacic acid as monomers. Subsequently, the PBPSS-75 composite electrolyte was prepared with this copolyester as the matrix and porous PI as support. Results show that long-carbon-chain sebacic acid effectively regulates polymer segment flexibility and free volume, synergistically enhancing ionic conductivity and interfacial mechanical stability with lithium metal. Experimental data indicate that PBPSS-75 composite electrolyte exhibits an ionic conductivity of up to 4.25 × 10^−5^ S cm^−1^ (30 °C), a lithium-ion transference number of 0.81, and an electrochemical stability window of 4.48 V (vs. Li/Li^+^). In LiFePO_4_//Li batteries, it maintains nearly 100% capacity retention after 300 cycles at 0.5 C, and achieves stable cycling for over 800 h in lithium symmetric cells. This study confirms that the combined strategy effectively addresses the conductivity-mechanical property trade-off of SPEs, providing theoretical guidance and technical reference for high-performance solid-state battery material design.

## 1. Introduction

As portable and highly reliable power sources, lithium-ion batteries (LIBs) have successfully superseded lead-acid and nickel-metal hydride batteries, finding extensive applications in electric vehicles (EVs), energy storage systems (ESSs), and consumer electronics [1,2,3]. While advancements in battery technology have promoted low-carbon and sustainable development, they have concurrently triggered widespread concerns regarding energy density bottlenecks and safety hazards [4,5,6]. All-solid-state lithium batteries (ASSLBs) are regarded as one of the most promising solutions to address these challenges. The core strategy involves replacing the flammable and explosive liquid electrolytes in traditional LIBs with intrinsically heat-resistant and non-flammable solid-state electrolytes, thereby significantly enhancing safety performance. Furthermore, the superior compatibility of solid-state electrolytes with high-energy-density lithium metal and alloy anodes enables the realization of higher energy densities at the cell level [7,8,9,10,11].

As the pivotal component of ASSLBs, solid-state electrolytes are generally categorized into inorganic solid electrolytes (ISEs) and solid polymer electrolytes (SPEs) [12,13,14]. ISEs encompass oxide-based (e.g., Li_7_La_3_Zr_2_O_12_, Li_1.3_Al_0.3_Ti_1.7_(PO4)_3_), sulfide-based (e.g., Li_10_GeP_2_S_12_), and halide-based electrolytes [15,16,17]. Although some ISEs achieve high room-temperature ionic conductivities up to 10^−2^ S cm^−1^, their practical application is often constrained by complex synthesis processes, large rigid interfacial resistance, and high sensitivity to moisture [18,19]. In contrast, SPEs have emerged as strong contenders for ASSLBs due to their facile processing, superior interfacial contact, and excellent thermal stability [20,21,22]. Currently, research on SPEs predominantly focuses on polyether-based polymers, represented by polyethylene oxide (PEO), where lithium-ion migration is facilitated through the complexation and dissociation of Li^+^ with ether-oxygen groups [23]. However, PEO-based electrolytes are restricted by low room-temperature ionic conductivity (10^−7^ S cm^−1^), limited lithium-ion transference numbers (<0.2), and narrow electrochemical stability windows (<3.9 V) [24,25,26,27]. Consequently, research attention has increasingly shifted toward polyester-based solid polymer electrolytes. For instance, Zhou et al. [28] synthesized five polyoxalates (POEs) via the transesterification polycondensation of alkyl diols with varying carbon chain lengths, revealing that POEs composed of odd-numbered carbon diols exhibited universally higher ionic conductivities due to reduced ordered intermolecular stacking. Subsequently, Xie et al. [29] synthesized 23 fluorinated linear polyesters, reaffirming the critical role of molecular asymmetry and reduced inter-chain aggregation in facilitating lithium-ion migration.

Recently, cutting-edge research has provided new paradigms for overcoming the limitations of polymer electrolytes from both molecular and structural perspectives. At the molecular level, precise regulation of intermolecular interactions has proven crucial. For instance, mitigating the strong coordination between Li^+^ and ether oxygen, rather than merely reducing crystallinity, effectively boosts the room-temperature performance of poly(ethylene oxide)-based electrolytes [30]. Similarly, regulating hydrogen bonding in hybrid gel polymer electrolytes has shown great promise in optimizing phase separation and ionic mobility [31]. From a structural standpoint, integrating polymers with robust porous skeletons, such as ultra-thin electrospun fiber networks, can drastically enhance mechanical strength and stabilize the lithium anode while maintaining high ionic conductivity [32]. Furthermore, as the field advances toward greener technologies, developing water-processable and sustainable fabrication strategies—such as the design of biopolymer-based organohydrogel electrolytes [33]—represents a crucial forward-looking direction for next-generation solid-state devices.

Inspired by these advanced molecular and structural engineering concepts, this study proposes a synergistic optimization strategy to address the long-standing trade-off between ionic conductivity and mechanical strength. This approach combines “long-carbon-chain regulation” to enhance ion transport with a “porous polyimide (PI) support” to ensure mechanical integrity. In this work, we synthesized a novel linear random copolyester, designated as poly(1,3-propylene-co-1,4-butylene succinate-co-sebacate) (PBPSS-75), via direct melt polycondensation using 1,3-propanediol, 1,4-butanediol, succinic acid, and sebacic acid as monomers. Unlike previous binary systems, this high proportion of long-carbon-chain monomers is explicitly designed to effectively regulate segment flexibility and increase free volume, thereby facilitating Li^+^ transport, while the PI framework acts as a rigid skeleton to inhibit lithium dendrite growth. The resulting PBPSS-75 SPE exhibits a reasonable room-temperature ionic conductivity of 4.25 × 10^−5^ S cm^−1^ (30 °C), a high lithium-ion transference number of 0.81, a high tensile strength of 3.98 MPa (with an elongation of 52.1%), and a wide electrochemical stability window of 4.48 V (vs. Li/Li^+^). Comprehensive electrochemical testing demonstrates that the LiFePO_4_/PBPSS-75 SPE/Li batteries maintain nearly 100% capacity retention after 300 cycles at 0.5 C, while lithium symmetric cells achieve stable cycling for over 800 h. These results validate the efficacy of our combined strategy in developing high-performance SPEs for high-safety energy storage systems.

## 2. Materials and Methods

### 2.1. Raw Materials

Materials 1,3-Propanediol, 1,4-butanediol, succinic acid, sebacic acid, and hydroquinone were of analytical grade (AR) and purchased from Shanghai Aladdin Biochemical Technology Co., Ltd. (Shanghai, China). Tetrabutyl titanate (TBT) and N-methyl-2-pyrrolidone (NMP) (battery grade) were obtained from Shanghai Macklin Biochemical Co., Ltd. (Shanghai, China). Phosphorous acid, chloroform, acetonitrile, methanol, and tetrahydrofuran (THF) were all analytical grade reagents supplied by Chengdu Kelong Chemical Co., Ltd. (Chengdu, China).

For battery assembly, Lithium iron phosphate (LFP, LiFePO_4_), conductive carbon black (Super-P), and polyvinylidene fluoride (PVDF 5130) were provided by Guangdong Canrd New Energy Technology Co., Ltd. (Dongguan, China). Lithium metal foils (diameter: 16 mm) were purchased from Tianjin China Energy Lithium Co., Ltd. (Tianjin, China). The porous polyimide (PI) nanofiber membrane (thickness: ~17 μm) was purchased from Jiangxi Xiancai Nanofiber Technology Co., Ltd. (Nanchang, China). All chemicals were used as received without further purification.

### 2.2. Synthesis of Copolyesters

**Scheme 1:**
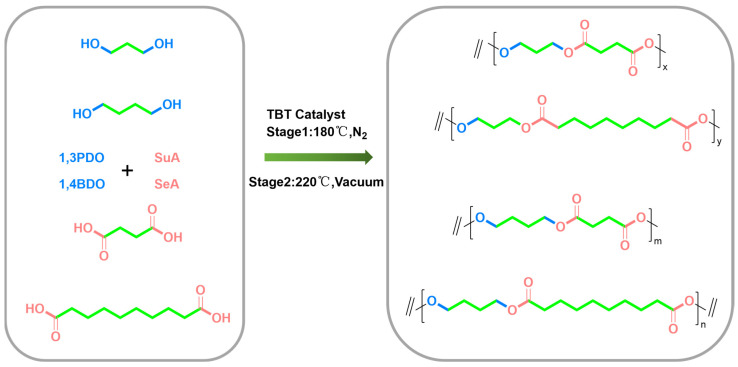
Synthetic route and reaction mechanism of the poly(1,3-propylene-co-1,4-butylene succinate-co-sebacate) (PBPSS) copolyester via two-step melt polycondensation.

The synthesis of the copolyester PBPSS-75 (poly(1,3-propylene-co-1,4-butylene succinate-co-sebacate)) is described as a representative example. The monomers 1,3-PDO, 1,4-BDO, SuA, and SeA were charged into a three-necked round-bottom flask equipped with a mechanical stirrer and a reflux condenser at a molar ratio of diols to diacids was set at 1.1:1 (specifically, n_1,3-PDO_:n_1,4-BDO_:n_SuA_:n_SeA_ = 1.1:1.1:0.5:1.5). Antioxidants, including phosphorous acid (0.04 wt%) and hydroquinone (0.01 wt%), were added to the mixture. The reaction system was purged with nitrogen (N_2_) three times to remove oxygen. The synthesis proceeded in two stages. First, the mixture was heated to 180 °C in an oil bath and maintained for 2 h under a continuous nitrogen flow to facilitate the esterification reaction. Subsequently, the temperature was cooled to below 100 °C, and the catalyst tetrabutyl titanate (TBT, 0.01 wt%) was added. The temperature was then increased to 220 °C under reduced pressure (vacuum) for the polycondensation stage. The reaction continued for 3–4 h until the “Weissenberg effect” (rod-climbing phenomenon) was observed. The detailed chemical reactions involved in both the esterification and polycondensation stages are schematically illustrated in Scheme 1. By adjusting the proportion of long-carbon-chain sebacic acid, a series of PBPSS copolyester (PBPSS-0, 25, 50, 75, and 100) with different monomer feed molar ratios were prepared, as detailed in Table 1.

After cooling to room temperature, the crude product was dissolved in chloroform (CHCl_3_) and precipitated in excess cold methanol to remove oligomers and unreacted monomers. Finally, the precipitate was collected by filtration and dried in a vacuum oven at 80 °C for 24 h.

### 2.3. Preparation of PI-Supported Composite Electrolytes

To prepare the composite electrolyte, the synthesized PBPSS-75 copolyester and LiTFSI were dissolved in a binary mixed solvent of chloroform and acetonitrile (CHCl_3_:CH_3_CN = 3:1, *v*/*v*) with a polymer-to-salt weight ratio of 1:0.4. The resulting homogeneous solution was drop-cast onto a porous polyimide (PI) nanofiber membrane to ensure thorough impregnation of the polymer matrix into the porous support. The wet composite membrane was then dried in a vacuum oven at 80 °C for 8 h to remove the residual solvent. Finally, the obtained composite electrolyte (denoted as PBPSS-75 SPE) was punched into disks with a diameter of 19 mm and stored in an argon-filled glove box (H_2_O < 0.1 ppm, O_2_ < 0.1 ppm).

### 2.4. Cathode Preparation and Cell Assembly

The LiFePO_4_ cathodes were prepared by mixing the active material, conductive agent (Super-P), and binder (PVDF) in a weight ratio of 8:1:1. The average mass loading of the active material was approximately [1.0–2.0] mg cm^−2^. The LiFePO_4_ cathode/SPE membrane/Li metal anode CR2032 coin cells were assembled in an argon-filled glove box. Post-assembly, the cells were aged in a thermostat oven at 50 °C for 12 h. This thermal annealing process leverages the rheological properties and segmental motion of the polymer matrix at elevated temperatures to promote in situ conformal contact between the electrolyte and the electrodes, thereby minimizing the interfacial impedance.

### 2.5. Material Characterization

The chemical structures and compositions of the polymers were characterized by Fourier transform infrared spectroscopy (FTIR, Nicolet iS20, Thermo Fisher Scientific, Waltham, MA, USA) and nuclear magnetic resonance spectroscopy (NMR, Quantum-I Plus 600 MHz, Wuhan Zhongke-Oxford NMR Technology Co., Ltd., Wuhan, China). Specifically, FTIR spectra were recorded in attenuated total reflection (ATR) mode over a wavenumber range of 4000–400 cm^−1^. The ^1^H NMR spectra were acquired using deuterated chloroform (CDCl_3_) as the solvent. The thermal stability and phase transition behaviors were evaluated via thermogravimetric analysis (TGA) and differential scanning calorimetry (DSC). TGA measurements were performed on a Mettler TGA/DSC1 instrument (Greifensee, Switzerland) from 30 to 800 °C at a heating rate of 10 °C min^−1^ under a nitrogen atmosphere. DSC measurements were conducted using a NETZSCH DSC300 Caliris instrument(Selb, Germany). To eliminate thermal history, a “heating–cooling–heating” protocol was employed (30 °C→200 °C→−100 °C→200 °C), with both heating and cooling rates set at 10 °C min^−1^, the samples were hermetically sealed in pure aluminum pans for the DSC measurements.

### 2.6. Electrochemical Measurements

Electrochemical measurements were performed using a CHI660E electrochemical workstation (Shanghai Chenhua Instrument Co., Ltd., Shanghai, China), while battery cycling tests were conducted on a LAND CT3001A battery testing system (Wuhan LAND Electronic Co., Ltd., Wuhan, China). To ensure the reliability and reproducibility of the data, key electrochemical measurements were performed on at least three independent parallel samples (*n* ≥ 3). Representative curves are presented in the figures. Various cell configurations were assembled to evaluate specific electrochemical properties:

1. Ionic Conductivity: The ionic conductivity was measured using SS/SPE/SS blocking cells (where SS denotes stainless steel) via electrochemical impedance spectroscopy (EIS). The EIS spectra were recorded in the frequency range of 1 Hz to 10^6^ Hz with an AC amplitude of 5 mV at [temperatures of 30, 40, 50, 60, and 70 °C].
2. Electrochemical Stability Window: The electrochemical stability window was determined by linear sweep voltammetry (LSV) using Li/SPE/SS cells. The voltage was scanned from 2.0 to 6.0 V (vs. Li/Li^+^) at a scan rate of 0.1 mV s^−1^.
3. Lithium-ion Transference Number (*t*_Li+_): The *t*_Li+_ was evaluated in Li/SPE/Li symmetric cells using the chronoamperometry (CA) method combined with EIS. A DC polarization voltage of 5 mV was applied for 3600 s to reach a steady-state current. EIS profiles were recorded before and after the polarization process.
4. Battery Performance: LiFePO_4_/SPE/Li cells were assembled to evaluate the cycling stability and rate capability. Galvanostatic charge/discharge (GCD) tests were performed within a voltage range of 2.7–4.0 V (vs. Li/Li^+^) at various C-rates at 25 °C.

## 3. Results and Discussion

### 3.1. Structural Characterization of PBPSS Copolyesters

**Figure 1:**
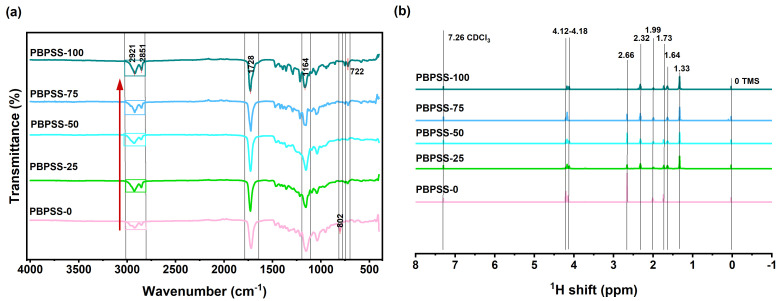
Chemical structure characterization of the poly(1,3-propylene-co-1,4-butylene succinate-co-sebacate) (PBPSS) copolymers: (**a**) Fourier transform infrared (FTIR) spectra of PBPSS with different sebacic acid contents; (**b**) Proton nuclear magnetic resonance (^1^H NMR) spectra of the synthesized copolymers (Solvent: CDCl_3_).

The chemical compositions and structures of the synthesized PBPSS copolymers were determined by FTIR and ^1^H NMR spectroscopies. Figure 1a presents the FTIR spectra of the PBPSS series (PBPSS-0 to PBPSS-100). All polymers exhibit similar characteristic peaks, confirming the existence of the polyester backbone. Specifically, the bands at 2921 and 2851 cm^−1^ are assigned to the asymmetric and symmetric stretching vibrations of methylene groups (-CH_2_-), respectively. From PBPSS-0 to PBPSS-100, the transmittance of the methylene (-CH_2_-) characteristic peaks shows a gradual decreasing trend, indicating a concomitant rise in the absorbance of methylene groups in the system. This characterization phenomenon is consistent with the trend that the content of methylene units in the system increases progressively with the introduction of long-chain sebacic acid. The intense peak at 1728 cm^−1^ corresponds to the C=O stretching vibration of the ester group, while the peak at 1164 cm^−1^ is attributed to the C-O-C=O stretching vibration. These characteristic peaks confirm the successful synthesis of the polyester structure. In the fingerprint region, structural variations due to different monomer ratios are clearly observed. The peak at 802 cm^−1^, ascribed to the characteristic vibration of the succinate segment, shows a low transmittance in PBPSS-0, and its transmittance gradually increases with the incorporation of sebacic acid. Conversely, the peak at 722 cm^−1^, attributed to the methylene rocking vibration of the long carbon chain [-(CH_2_)*_n_*-, *n* ≥ 4] in the sebacate segment, is absent in PBPSS-0. This peak appears in PBPSS-25, and its transmittance gradually decreases as the sebacic acid content increases up to PBPSS-100 [34,35]. These FTIR features demonstrate that the chain structure of the random copolymers was successfully regulated by adjusting the feed ratio.

Figure 1b shows the ^1^H NMR spectra of the copolymers (Solvent: CDCl_3_). The proton signals are well-resolved and perfectly match the chemical structure of the copolyester consisting of 1,3-propanediol (1,3-PDO), 1,4-butanediol (1,4-BDO), succinic acid, and sebacic acid units.

1. Diol Units: The intense multiple peak centered at 4.16 ppm is attributed to the overlapping α-methylene protons (-OCH_2_-) of both 1,3-PDO and 1,4-BDO segments, as their chemical environments are highly similar. The distinct peak at 1.99 ppm is assigned to the central β-methylene protons (-CCH_2_C-) of the 1,3-PDO unit. Crucially, the signal at 1.73 ppm corresponds to the central methylene protons (-CH_2_CH_2_-) of the 1,4-BDO unit, which partially overlaps with the β-methylene protons of the sebacate segment.
2. Diacid Units: Regarding the dicarboxylic acid segments, the peak at 2.66 ppm corresponds to the α-methylene protons (-OOC-CH_2_-) of the succinate unit; its intensity decreases with reducing succinic acid content. The sebacate segment displays characteristic peaks at 2.32 ppm (α-CH_2_), 1.64 ppm (part of β-CH_2_), and 1.33 ppm (bulk CH_2_).

Combined with the FTIR and ^1^H NMR results, it is confirmed that the PBPSS random copolyesters were successfully synthesized, and the actual composition aligns well with the feed ratio.

### 3.2. Thermal Stability and Mechanical Properties

**Figure 2:**
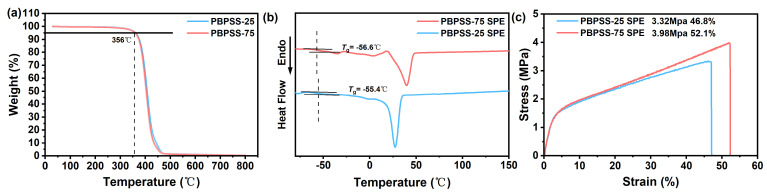
Thermal and mechanical properties: (**a**) Thermogravimetric analysis (TGA) curves; (**b**) Differential scanning calorimetry (DSC) thermograms; (**c**) Tensile stress–strain curves.

Thermogravimetric analysis (TGA) was conducted under a nitrogen atmosphere to evaluate the thermal stability of the synthesized copolymers. As illustrated in Figure 2a, PBPSS-25 and PBPSS-75 were selected as representative samples for this measurement. Both polymers exhibited similar thermal degradation behaviors, with a high onset decomposition temperature (*T_d_*, *5%*) of approximately 356 °C. The main degradation step was completed at around 470 °C. This excellent thermal stability is sufficient to satisfy the safety and operational requirements of most solid-state lithium batteries [36,37,38]. Differential scanning calorimetry (DSC) was employed to investigate the thermal transition behaviors and chain flexibility of the synthesized electrolytes. As presented in Figure 2b, both copolymers exhibit low glass transition temperatures (*T_g_*) well below room temperature. Notably, PBPSS-75 possesses the lowest *T_g_* of −56.6 °C, which is lower than that of PBPSS-25 (−55.4 °C). This reduction in *T_g_* is attributed to the introduction of longer methylene segments from the sebacic acid units, which act as internal plasticizers to enhance the flexibility of the polymer chains. Generally, a lower *T_g_* signifies more vigorous segmental motion and larger free volume within the polymer matrix. This enhanced chain flexibility in PBPSS-75 effectively facilitates the dissociation of lithium salts and the migration of Li^+^ ions, which aligns perfectly with the ionic conductivity results where PBPSS-75 SPE exhibits the highest conductivity [39,40]. Mechanical integrity is a prerequisite for SPEs to physically suppress lithium dendrite growth and accommodate electrode volume changes [41,42]. To compensate for the insufficient mechanical strength of the intrinsic polymer matrix, a porous polyimide (PI) membrane was employed as a rigid scaffold to fabricate the PBPSS-25 SPE and PBPSS-75 SPE. The representative stress–strain curves are depicted in Figure 2c. Reinforced by the PI host, PBPSS-75 SPE exhibits a mechanically self-standing nature with a tensile strength of 3.98 MPa and an elongation at break of 52.1%, compared to 3.32 MPa and 46.8% for PBPSS-25 SPE. Although lower than that of commercial polyolefin separators, this mechanical strength is significantly superior to that of conventional PEO-based electrolytes (~1–2 MPa) [43,44]. More importantly, the adequate ductility (elongation > 50%) ensures intimate interfacial contact with the electrodes during repeated charge/discharge cycles, while the PI reinforcement provides a safeguard against potential dendrite penetration. Corresponding thermal (TGA/DSC) and mechanical characterizations for other compositions (PBPSS-0, 50, and 100) are also included in Figure S2 (Supporting Information) for a complete series comparison.

### 3.3. Ion Transport Properties and Electrochemical Stability

**Figure 3:**
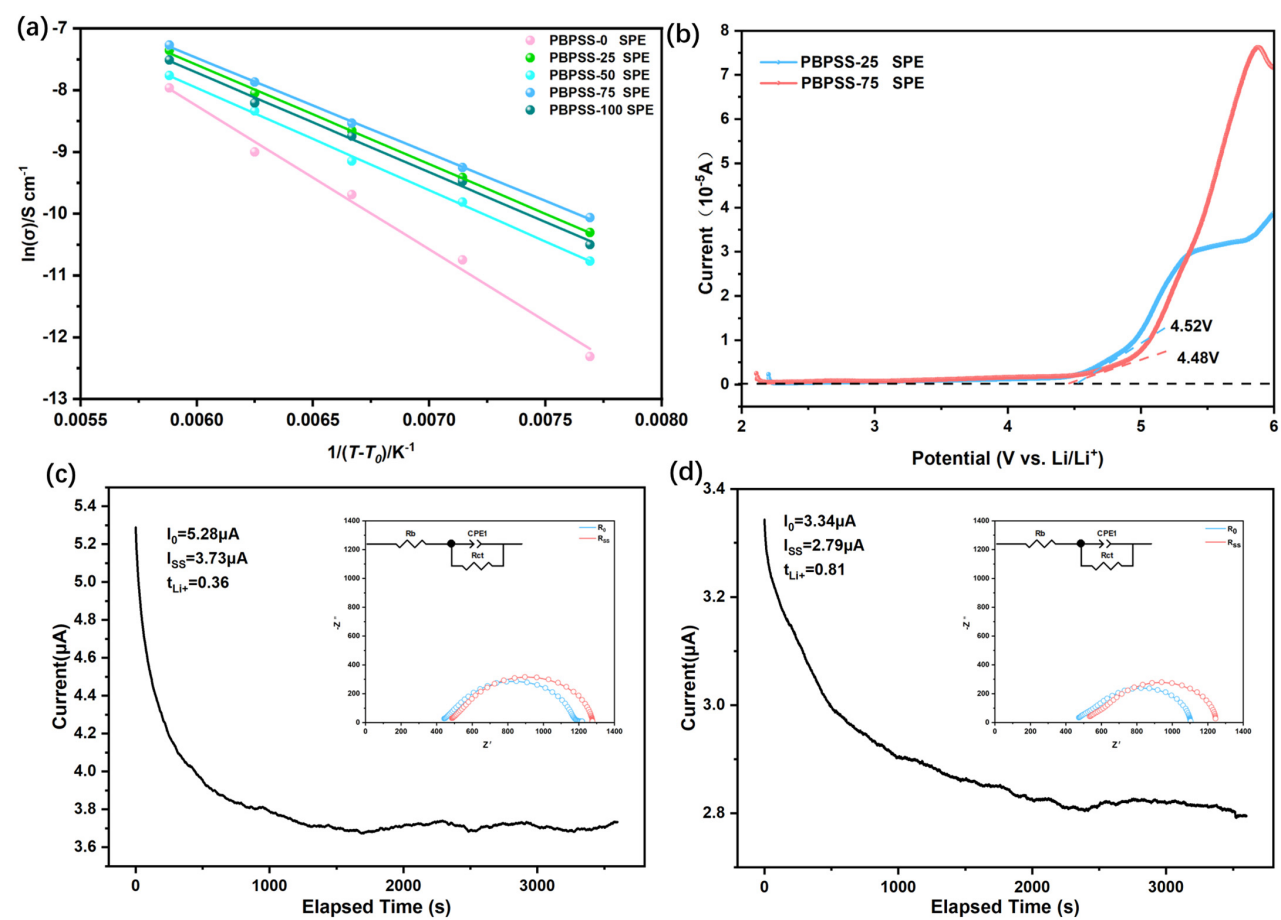
Electrochemical characterization of PBPSS electrolytes: (**a**) Vogel–Tammann–Fulcher (VTF) plots of ionic conductivity for PBPSS SPES with different compositions; (**b**) Linear sweep voltammetry (LSV) curves of PBPSS-25 SPE and PBPSS-75 SPE; (**c**,**d**) Chronoamperometry profiles of symmetrical Li/SPE/Li cells for (**c**) PBPSS-25 SPE and (**d**) PBPSS-75 SPE to determine the lithium-ion transference number (*t*_Li+_). Insets show the AC impedance spectra before and after polarization.

Ionic conductivity is a decisive parameter determining the performance of solid-state electrolytes. Figure 3a presents the temperature dependence of ionic conductivity for the PBPSS series (PBPSS-0 SPE to PBPSS-100 SPE). As expected, all electrolytes exhibit a well-defined linear relationship, following typical VTF behavior. This indicates that ion transport within the system is a thermally activated process primarily governed by the segmental motion of polymer chains [45,46]. At 30 °C, the ionic conductivities of the series were calculated. Notably, PBPSS-75 SPE exhibits the highest room-temperature ionic conductivity of (4.25 ± 0.15) × 10^−5^ S cm^−1^, which is significantly higher than that of PBPSS-0 SPE (4.48 × 10^−6^ S cm^−1^) and other counterparts. The corresponding activation energies (*Ea*), calculated from the slopes of the VTF plots, are 0.2, 0.139, 0.143, 0.133, and 0.139 eV for PBPSS-0, 25, 50, 75, and 100 SPEs, respectively. PBPSS-75 SPE possesses the lowest activation energy (0.133 eV), suggesting the lowest energy barrier for ion hopping. The VTF fit parameters are given in the Supporting Information Table S1. This result is highly consistent with the previous DSC analysis, where PBPSS-75 SPE showed the lowest *T_g_*. The enhanced chain flexibility and increased free volume in PBPSS-75 SPE facilitate the dissociation of lithium salts and the rapid migration of Li^+^ ions. The electrochemical stability window (ESW) of the electrolytes was evaluated by linear sweep voltammetry (LSV) at room temperature Figure 3b. PBPSS-25 SPE and PBPSS-75 SPE demonstrate anodic oxidation voltages of 4.52 V and 4.48 V (vs. Li/Li^+^), respectively, indicating good high-voltage stability [47]. To further quantify the contribution of lithium ions to the total conductivity, the lithium-ion transference number (*t*_Li+_) was determined using the Bruce–Vincent method [48]. Figure 3c and Figure 3d display the chronoamperometry profiles and the initial/steady-state impedance spectra (insets) for PBPSS-25 SPE and PBPSS-75 SPE symmetric cells, respectively. The calculated *t*_Li+_ for PBPSS-25 SPE is 0.36, whereas PBPSS-75 SPE achieves a significantly improved value of 0.81. This superior cation transport selectivity is hypothesized to stem from the synergistic effect of the polymer matrix and the PI support: the abundant ester groups in PBPSS-75 SPE facilitate Li+ hopping, while the tortuous porous structure of the PI framework and its interaction with the polymer matrix are believed to provide steric hindrance, which could potentially impede migration of bulky TFSI-anions. Although further direct evidence is required to fully elucidate this specific ionic decoupling mechanism in future studies, the high *t*_Li+_ of PBPSS-75 SPE is instrumental in mitigating concentration polarization during long-term cycling, thereby contributing to the suppression of lithium dendrite growth.

### 3.4. Evaluation of Interfacial Stability Against Lithium Metal

**Figure 4:**
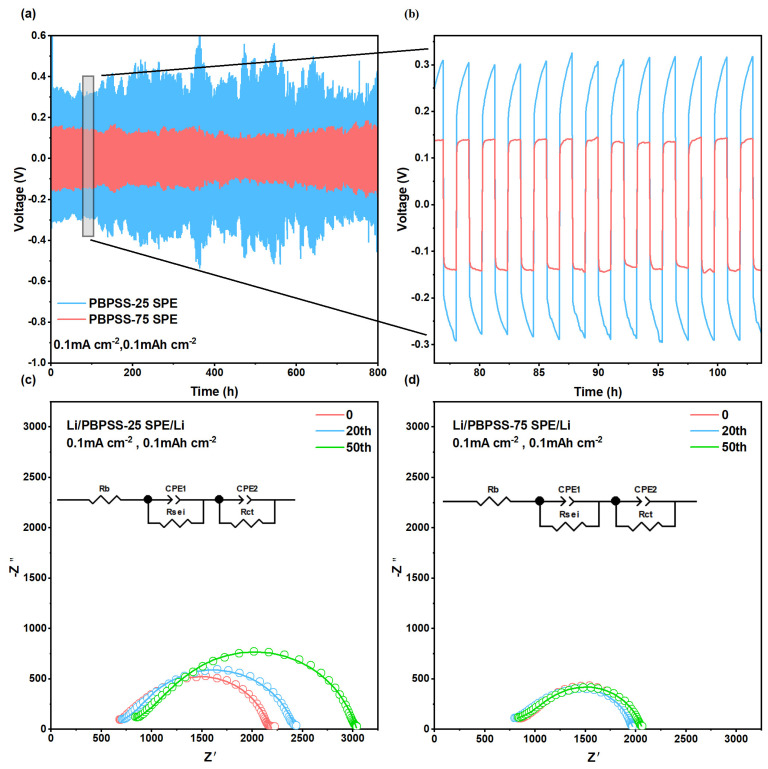
Evaluation of the interfacial stability against lithium metal: (**a**) Galvanostatic cycling performance of Li/PBPSS-25 SPE/Li and Li/PBPSS-75 SPE/Li symmetric cells at a current density of 0.1 mA cm^−2^ and an areal capacity of 0.1 mAh cm^−2^; (**b**) Detailed voltage profiles at selected cycle; (**c**) and (**d**) Evolution of electrochemical impedance spectra (EIS) for the PBPSS-25 SPE and PBPSS-75 SPE cells during cycling, respectively.

Figure 4a presents the long-term galvanostatic cycling voltage profiles of lithium symmetric cells assembled with PBPSS-25 SPE and PBPSS-75 SPE at a current density of 0.1 mA cm^−2^ with a fixed areal capacity of 0.1 mAh cm^−2^. The Li/PBPSS-25 SPE/Li cell exhibits severe voltage fluctuations during cycling, which are typically attributed to uneven lithium deposition-induced dendrite growth and the subsequent micro-shorts or interfacial instability. In sharp contrast, the Li/PBPSS-75 SPE/Li cell demonstrates superior cycling stability, operating steadily for over 800 h without any signs of short circuit. The detailed voltage profiles in Figure 4b further reveal that the overpotential of the PBPSS-75 SPE cell is 140 mV, significantly lower than that of the PBPSS-25 SPE cell (~300 mV). This low and stable overpotential is primarily attributed to the high ionic conductivity and remarkably high lithium-ion transference number (0.81) of PBPSS-75 SPE, which facilitate fast ion transport and effectively mitigate concentration polarization during charge/discharge processes, thereby inducing uniform lithium deposition.

To quantitatively unravel the interfacial evolution mechanism, the EIS spectra at different cycling stages (0, 20th, and 50th cycles) were fitted using an equivalent circuit model. As shown in Figure 4c, the Li/PBPSS-25 SPE/Li cell exhibits a continuous and severe degradation. The bulk resistance (*R*_b_) increased from 565.4 Ω (0 cycle) to 709.6 Ω (50th cycle), suggesting contact loss at the interface. More critically, the SEI resistance (*R*_sei_) surged dramatically from 187.9 Ω at the 20th cycle to 480.8 Ω at the 50th cycle. This increase of over 150% implies that the fragile SEI layer, unsupported by a rigid matrix, underwent repeated fracture and reconstruction, leading to the continuous thickening of the passivation layer. Meanwhile, the charge transfer resistance (*R*_ct_) remained at a high level (>1600 Ω), indicating sluggish interfacial kinetics. In contrast, the Li/PBPSS-75 SPE/Li cell, Figure 4d, demonstrates a superior “activation-stabilization” behavior. Initially, the *R*_ct_ dropped significantly from 1370.7 Ω (0 cycle) to 924.9 Ω (20th cycle), reflecting an effective electrochemical activation process that improves interfacial wetting. Most notably, the interface entered a highly stable state in subsequent cycles. From the 20th to the 50th cycle, the *R*_sei_ remained virtually constant (424.8 Ω vs. 435.2 Ω), and the *R*_ct_ showed only negligible fluctuation (924.9 Ω vs. 988.7 Ω). This “freezing-like” impedance characteristic strongly corroborates that the mechanically reinforced PI scaffold effectively suppresses dendrite growth and volume expansion, facilitating the formation of a robust, thin, and kinetically stable SEI layer.

To visually confirm this, SEM images of lithium anodes after 100 h of cycling were obtained (Figure S1, Supporting Information). The lithium surface in the liquid electrolyte shows severe pulverization and mossy dendrites, Figure S1a. In contrast, PBPSS-75 SPE maintains a remarkably smooth and dense interface, Figure S1c, while PBPSS-25 SPE shows minor dendritic protrusions (Figure S1b). These observations suggest that the high-modulus PI scaffold and improved *t*_Li+_ (0.81) of PBPSS-75 SPE play a positive role in regulating uniform lithium deposition and mitigating dendrite penetration.

### 3.5. Electrochemical Performance of LiFePO_4_//Li Cells

**Figure 5:**
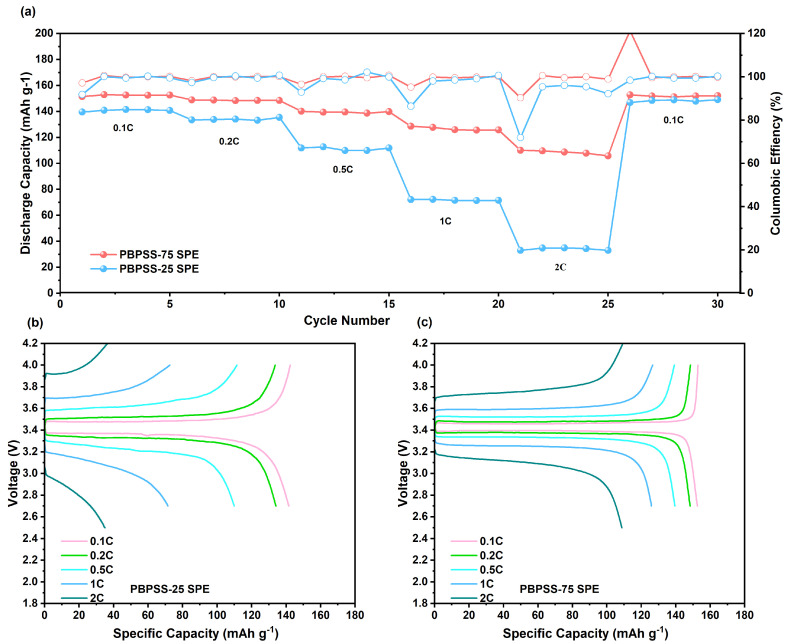
Rate capability of the assembled solid-state cells: (**a**) Rate performance comparison between LiFePO_4_/PBPSS-25 SPE/Li and LiFePO_4_/PBPSS-75 SPE/Li cells at 25 °C (**b**,**c**) Galvanostatic charge/discharge profiles of (**b**) PBPSS-25 SPE and (**c**) PBPSS-75 SPE at various C-rates.

To evaluate the electrochemical kinetics under practical operating conditions, the rate capability of the LiFePO_4_/SPE/Li cells was investigated at 25 °C, as shown in Figure 5a. The figure displays the specific discharge capacities of the cells at various C-rates ranging from 0.1 C to 2 C. The cell employing the PBPSS-75 SPE exhibits superior specific capacity compared to the PBPSS-25 SPE counterpart across all tested current densities. Specifically, the PBPSS-75 SPE cell delivers reversible specific capacities of 152.4, 148.6, 139.5, 126.7, and 108.4 mAh g^−1^ at 0.1 C, 0.2 C, 0.5 C, 1 C, and 2 C, respectively. Even at a high rate of 2 C, a respectable capacity retention of 71.1% (relative to 0.1 C) is achieved. Upon returning the current density to 0.1 C, the capacity recovers almost completely, demonstrating excellent reversibility and structural stability. In contrast, the PBPSS-25 SPE cell suffers from severe capacity decay at elevated rates, indicating significant kinetic sluggishness within the electrolyte. Figure 5c further illustrates the typical galvanostatic charge/discharge voltage profiles of the PBPSS-75 SPE cell. Even at high current densities, the cell maintains flat and well-defined voltage plateaus without obvious distortion. Notably, the voltage polarization (the difference between charge and discharge plateaus) exhibits only a marginal increase as the C-rate rises. This superior rate capability and low polarization are primarily attributed to the remarkably high lithium-ion transference number (*t*_Li+_ 0.81) and favorable ionic conductivity of PBPSS-75 SPE. These properties effectively mitigate concentration polarization during rapid charge/discharge processes, ensuring fast Li^+^ transport between the cathode and anode.

**Figure 6:**
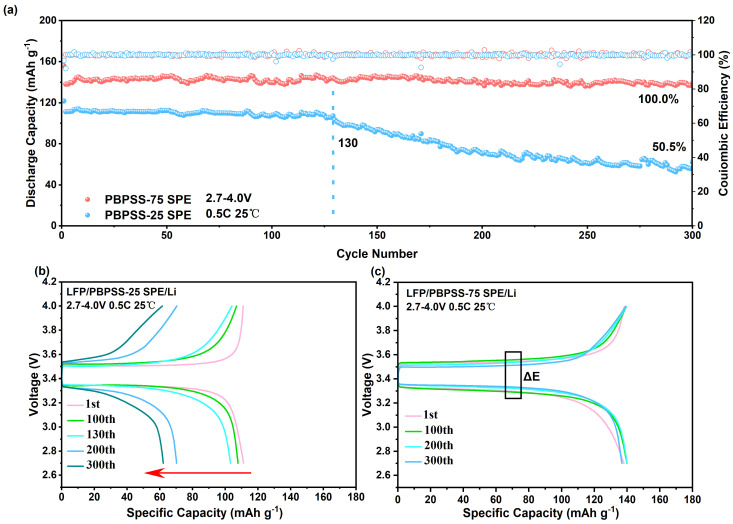
Long-term cycling performance of the assembled solid-state cells: (**a**) Discharge capacity and Coulombic efficiency of LiFePO_4_/PBPSS-25 SPE/Li and LiFePO_4_/PBPSS-75 SPE/Li at 0.5 C; (**b**,**c**) Typical charge/discharge voltage profiles of the (**b**) PBPSS-25 SPEand (**c**) PBPSS-75 SPE based cells at the 1st, 100th,130th, 200th, and 300th cycles.

To evaluate the operational stability under practical conditions, the long-term cycling performance of the cells was investigated at 0.5 C/0.5 C and 25 °C, as shown in Figure 6a. The LiFePO_4_/PBPSS-75 SPE/Li cell exhibited exceptional durability, delivering a high initial specific discharge capacity of 139.5 mAh g^−1^. After 300 deep cycles, the capacity was firmly retained at 139.4 mAh g^−1^, corresponding to a capacity retention approaching 100%. In contrast, the control LiFePO_4_/PBPSS-25 SPE/Li cell displayed inferior stability with a lower initial capacity of 111.2 mAh g^−1^. Although it maintained stable operation initially, a distinct turning point occurred after the 130th cycle, leading to rapid capacity decay. By the 300th cycle, the retention rate dropped to only 50.5% (56.2 mAh g^−1^). Notably, during the initial ~100 cycles, the PBPSS-75 cell shows a slight capacity climb because its highly dynamic polymer chains effectively penetrate the porous cathode (electrochemical activation). Conversely, the continuous capacity decay of PBPSS-25 from the beginning indicates that severe interfacial polarization rapidly outpaces its sluggish wetting process. Furthermore, excluding formation cycles, PBPSS-75 delivers a remarkable average Coulombic efficiency of 99.89% (vs. 99.52% for PBPSS-25), confirming highly reversible lithium plating/stripping and superior interfacial stability.

To further unravel the interfacial evolution mechanisms, the galvanostatic charge/discharge voltage profiles at selected cycles (1st, 100th, 130th, 200th, and 300th) were scrutinized, Figure 6b,c. For the PBPSS-25 SPE cell, the voltage profiles exhibited a drastic shift to the left (capacity shrinkage) and a widening of voltage hysteresis after 130 cycles, Figure 6b. This indicates that PBPSS-25 contains a low content of long-chain sebacic acid units, resulting in inferior ionic conductivity and lithium-ion transference number as well as sluggish Li^+^ transport kinetics. This not only fails to suppress the accumulation of parasitic side reactions and dead lithium, leading to a drastic increase in the internal resistance of the cell, but also gives rise to a significant rise in the polarization potential of the electrochemical plateaus, as reflected by the widened gap between the charge and discharge plateaus and aggravated polarization, further verifying the insufficient ion transport efficiency and interfacial stability. Conversely, the PBPSS-75 SPE cell demonstrated a low polarization behavior, where the voltage profiles remained highly overlapped throughout the test Figure 6c. Notably, the polarization voltage (ΔE), calculated at a capacity of 70 mAh g^−1^, showed a decreasing trend upon cycling: 0.245 V (1st cycle) → 0.266 V (100th cycle) → 0.214 V (200th cycle) → 0.180 V (300th cycle). The reduced polarization is mainly ascribed to the introduction of long-chain sebacic acid units, which effectively enhances the ionic conductivity and Li^+^ transference number, thus optimizing ion transport dynamics; meanwhile, the mechanically reinforced PI scaffold maintains interfacial contact integrity. The synergistic effect contributes to the formation of a stable and low-resistance interface during cycling.

It should be objectively noted that while the room-temperature ionic conductivity of the PBPSS-75 SPE (4.25 × 10^−5^ S cm^−1^ at 30 °C) is sufficient for stable battery operation, it remains reasonable compared to some state-of-the-art polymer or composite electrolytes. This limitation originates from the intrinsic segmental mobility constraints of the solid-state polyester matrix at room temperature. However, this modest absolute conductivity is effectively compensated by the remarkably high lithium-ion transference number (0.81) of the PBPSS-75 SPE, which predominantly carries the ionic current via effective Li^+^ cations and significantly mitigates concentration polarization during long-term cycling. To comprehensively evaluate the competitiveness of the designed electrolyte under practical conditions, the overall performance of the PBPSS-75 SPE was compared with recently reported state-of-the-art polymer electrolytes, as summarized in Table 2.

## 4. Conclusions

In summary, a high-performance composite solid polymer electrolyte (PBPSS-75 SPE) was successfully developed via a synergistic optimization strategy combining long-carbon-chain structural regulation and porous polyimide (PI) reinforcement. The experimental results demonstrate that the introduction of long-chain sebacic acid units effectively reduced the glass transition temperature (*T_g_*) of the copolyester to −56.6 °C, thereby enhancing chain flexibility and promoting lithium-ion migration. Meanwhile, the rigid porous PI support endowed the electrolyte with a mechanically self-standing nature (tensile strength of 3.98 MPa) and improved thermal stability, effectively resolving the intrinsic trade-off between ionic conductivity and mechanical strength in traditional SPEs. Consequently, the optimized PBPSS-75 SPE exhibited a reasonable room-temperature ionic conductivity of 4.25 × 10^−5^ S cm^−1^ and a remarkably high lithium-ion transference number of 0.81, attributed to the anion-immobilizing effect of the composite framework. These properties enabled the LiFePO_4_/PBPSS-75 SPE/Li solid-state batteries to achieve exceptional cycling stability with nearly 100% capacity retention after 300 cycles at 0.5 C, as well as dendrite-free lithium plating/stripping for over 800 h. This work confirms that the dual-regulation strategy of “molecular design + structural reinforcement” is a highly effective approach for developing advanced SPEs, providing valuable theoretical insights and technical solutions for the practical application of high-energy-density and high-safety solid-state batteries.

## Figures and Tables

**Table 1:** Molar feed ratios of the monomers used for poly(1,3-propylene-co-1,4-butylene succinate-co-sebacate) (PBPSS)synthesis.

Sample	Feed Ratio (Molar Ratio)			
	1,3PDO	1,4BDO	SuA	SeA
PBPSS-0	1.1	1.1	2	0
PBPSS-25	1.1	1.1	1.5	0.5
PBPSS-50	1.1	1.1	1	1
PBPSS-75	1.1	1.1	0.5	1.5
PBPSS-100	1.1	1.1	0	2

Abbreviations: 1,3-PDO: 1,3-propanediol; 1,4-BDO: 1,4-butanediol; SuA: succinic acid; SeA: sebacic acid.

**Table 2:** Performance comparison between the PBPSS-75 SPE and other recently reported polymer electrolytes.

Polymer Matrix	Ionic Conductivity	Lithium-Ion Transference Number	Cell Cycling Stability	Ref.
PEO-based SPE	1.76 × 10^−4^ S cm^−1^ (35 °C)	0.49	100 cycles (Li|LFP, 0.1 C, 90.5% ret.)	[30]
PEGDA/PAN electrospun	8.80 × 10^−4^ S cm^−1^ (RT)	N/A	150 cycles (Li|LFP, 0.3 C, 87.7% ret.)	[32]
Poly(pentanediol adipate)	0.59 × 10^−4^ S cm^−1^ (RT)	N/A	300 cycles (Li|LFMP, 0.5 C, 95.0% ret.)	[29]
PDEGA-QSPE	2.30 × 10^−4^ S cm^−1^ (RT)	N/A	300 cycles (Li|LFP, 0.2 C, 92.5% ret.)	[49]
PBPSS-75 SPE/PI (This work)	(4.25 ± 0.15) × 10^−5^ S cm^−1^ (30 °C)	0.81	300 cycles (Li|LFP, 0.5 C, ~100.0% ret.)	This work

## Data Availability

The data presented in this study are available on request from the corresponding author.

## Supplementary Materials

The following supporting information can be downloaded at: https://www.mdpi.com/article/10.3390/polym18060685/s1, Figure S1. Scanning electron microscopy (SEM) images of the lithium metal surfaces harvested from symmetric cells after 50 cycles: (a) with liquid electrolyte, (b) with PBPSS-25 electrolyte, and (c) with PBPSS-75 electrolyte; Figure S2. Hermal and mechanical characterizations of PBPSS-0, PBPSS-50, and PBPSS-100. (a) TGA curves; (b) DSC thermograms; (c) Tensile stress–strain curves; Table S1. Fitting parameters of ionic conductivity for PBPSS electrolytes based on the VTF equation. Reference [50] are cited in the supplementary materials.
