# Supplementary material for: High-Performance Solid Polymer Electrolyte Constructed from Long-Chain Regulated Random Copolymers and Porous PI Composites
Source: Polymers (Basel). 2026 Mar 11;18(6):685. doi: 10.3390/polym18060685 (PMC13029849; doi:10.3390/polym18060685)
Supplement: Supplementary file 1 [file polymers-18-00685-s001.zip › polymers-4181156-supplementary.pdf]

Supplementary Materials

High-Performance Solid Polymer Electrolyte Constructed from Long-Chain Regulated Random Copolymers and Porous PI Composites

Qian Zhang [2], Mingyang Cao [1], Chenxia Tang [1], Yuqing Zhou [1] and Xiaoli Peng [1,3,*]

1 School of Materials and Energy, University of Electronic Science and Technology of China, Chengdu 611731, China; 202321030322@std.uestc.edu.cn (M.C.); 202211030527@std.uestc.edu.cn (C.T.); 202322030332@std.uestc.edu.cn (Y.Z.)

2 School of Integrated Circuit Science and Engineering (Exemplary School of Microelectronics), University of Electronic Science and Technology of China, Chengdu 611731, China; zq@uestc.edu.cn

3 Frontier Center of Energy Distribution and Integration, Tianfu Jiangxi Lab, No. 366, Laboratory Road, Chengdu 641419, China

* Correspondence: pxl@uestc.edu.cn

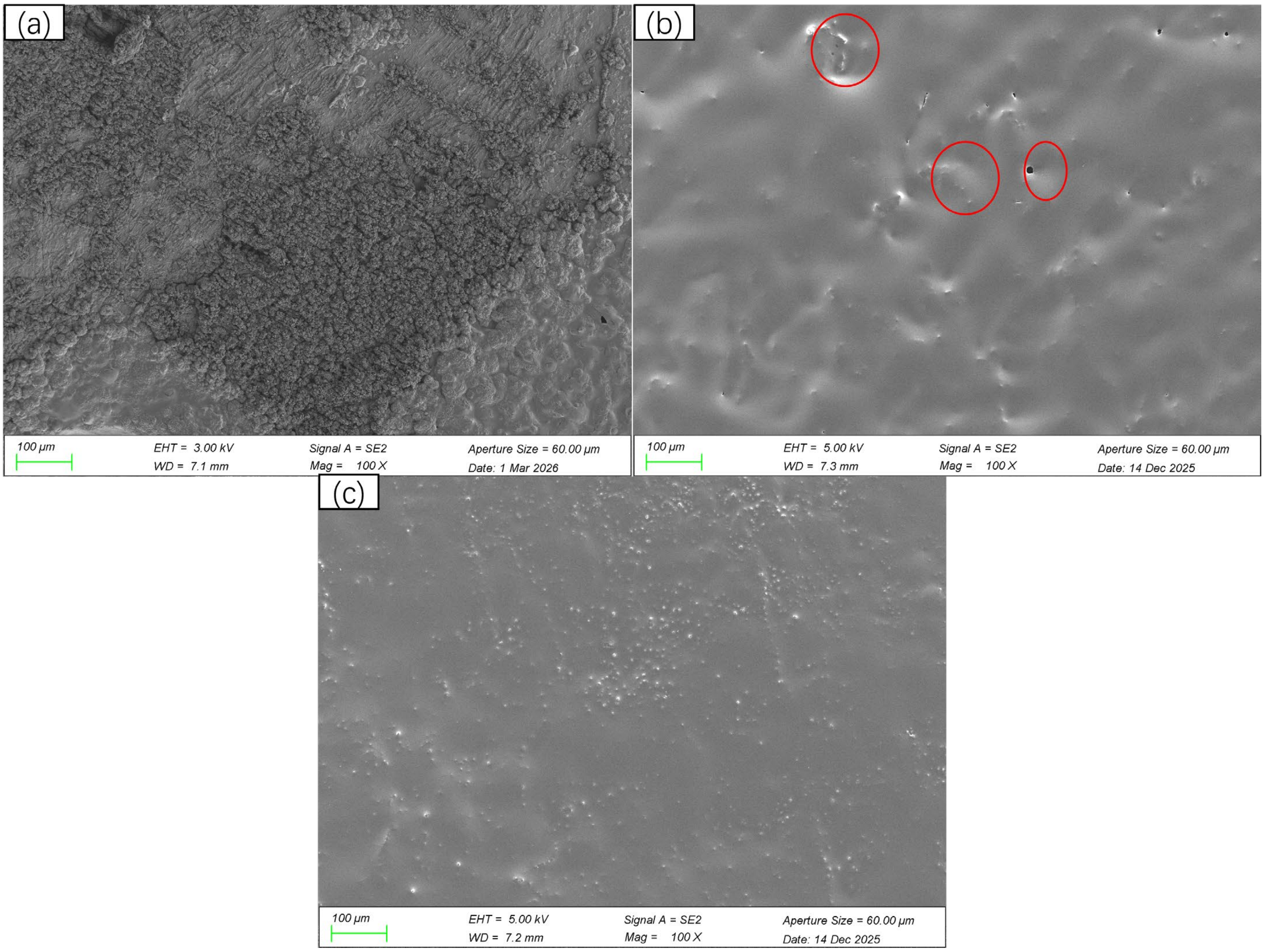

Figure S1. Scanning electron microscopy (SEM) images of the lithium metal surfaces harvested from symmetric cells after 50 cycles: (a) with liquid electrolyte, (b) with PBPSS-25 electrolyte, and (c) with PBPSS-75 electrolyte.

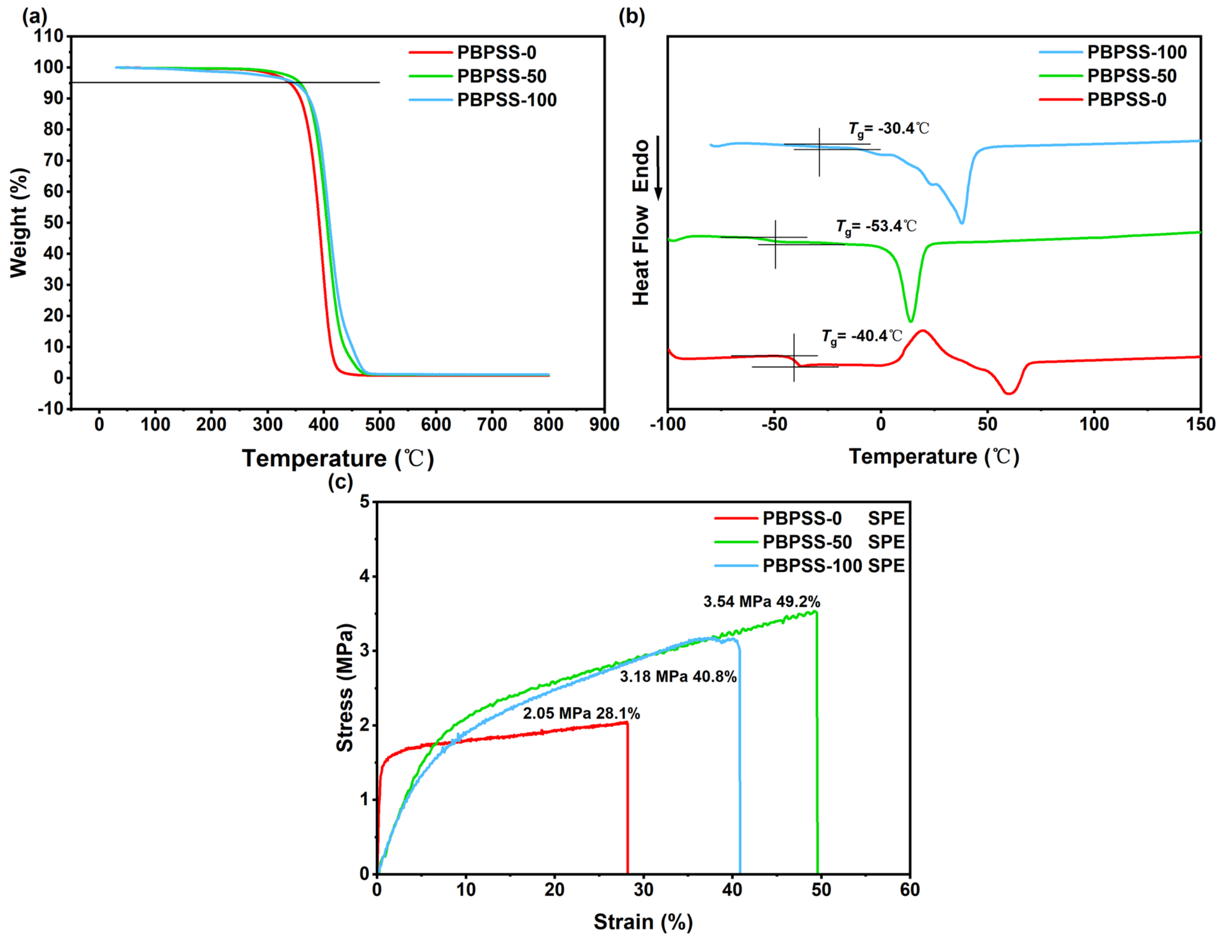

Figure S2. Hermal and mechanical characterizations of PBPSS-0, PBPSS-50, and PBPSS-100. (a) TGA curves; (b) DSC thermograms; (c) Tensile stress-strain curves.

Table S1. Fitting parameters of ionic conductivity for PBPSS electrolytes based on the VTF equation.

Sample	Slope [K]	R^2	B [kJ mol^{-1}]	Ea [eV]
PBPSS-0 SPE	-2323.296 ± 115.801	0.990	19.32 ± 0.96	0.200 ± 0.010
PBPSS-25 SPE	-1607.643 ± 29.906	0.999	13.37 ± 0.25	0.139 ± 0.003
PBPSS-50 SPE	-1658.358 ± 40.339	0.998	13.79 ± 0.34	0.143 ± 0.004
PBPSS-75 SPE	-1543.298 ± 19.338	0.999	12.83 ± 0.16	0.133 ± 0.002
PBPSS-100 SPE	-1611.300 ± 56.795	0.995	13.40 ± 0.47	0.139 ± 0.005

$$\sigma = A \cdot \exp\left(-\frac{B}{R(T-T_0)}\right) \quad \text{(Eq. S1)}$$

$$\ln(\sigma) = -\frac{B}{R} \cdot \frac{1}{T-T_0} + \ln(A) \quad \text{(Eq. S2)}$$

1. Petry, J.; Dietel, M.; Thelakkat, M. Semi-Interpenetrating Network Electrolytes Utilizing Ester-Functionalized Low T_g Polysiloxanes in Lithium-Metal Batteries. *Polymers* **2023**, *15*, 2459. https://doi.org/10.3390/polym15112459.
